# A modified method for CT radiomics region-of-interest segmentation in adrenal lipid-poor adenomas: a two-institution comparative study

**DOI:** 10.3389/fonc.2023.1086039

**Published:** 2023-04-19

**Authors:** Hanlin Zhu, Mengwei Wu, Peiying Wei, Min Tian, Tong Zhang, Chunfeng Hu, Zhijiang Han

**Affiliations:** ^1^ Department of Radiology, Hangzhou Ninth People’s Hospital, Hangzhou, China; ^2^ Department of Radiology, The Quzhou Affiliated Hospital of Wenzhou Medical University, Quzhou, China; ^3^ Department of Radiology, Affiliated Hangzhou First People’s Hospital, Zhejiang University School of Medicine, Hangzhou, China

**Keywords:** adrenal incidentaloma, adrenal neoplasm, radiomics, ROI, texture analysis

## Abstract

**Objective:**

This study aimed to investigate the application of modified region-of-interest (ROI) segmentation method in unenhanced computed tomography in the radiomics model of adrenal lipid-poor adenoma, and to evaluate the diagnostic performance using an external medical institution data set and select the best ROI segmentation method.

**Methods:**

The imaging data of 135 lipid-poor adenomas and 102 non-adenomas in medical institution A and 30 lipid-poor adenomas and 43 non-adenomas in medical institution B were retrospectively analyzed, and all cases were pathologically or clinically confirmed. The data of Institution A builds the model, and the data of Institution B verifies the diagnostic performance of the model. Semi-automated ROI segmentation of tumors was performed using uAI software, using maximum area single-slice method (MAX) and full-volume method (ALL), as well as modified single-slice method (MAX_E) and full-volume method (ALL_E) to segment tumors, respectively. The inter-rater correlation coefficients (ICC) was performed to assess the stability of the radiomics features of the four ROI segmentation methods. The area under the curve (AUC) and at least 95% specificity pAUC (Partial AUC) were used as measures of the diagnostic performance of the model.

**Results:**

A total of 104 unfiltered radiomics features were extracted using each of the four segmentation methods. In the ROC analysis of the radiomics model, the AUC value of the model constructed by MAX was 0.925, 0.919, and 0.898 on the training set, the internal validation set, and the external validation set, respectively, and the AUC value of MAX_E was 0.937, 0.931, and 0.906, respectively. The AUC value of ALL was 0.929, 0.929, and 0.918, and the AUC value of ALL_E was 0.942, 0.926, and 0.927, respectively. In all samples, the pAUCs of MAX, MAX_E, ALL, and ALL_E were 0.021, 0.025, 0.018, and 0.028, respectively.

**Conclusion:**

The diagnostic performance of the radiomics model constructed based on the full-volume method was better than that of the model based on the single-slice method. The model constructed using the ALL_E method had a stronger generalization ability and the highest AUC and pAUC value.

## 1 Introduction

Incidentally detected adrenal masses, called adrenal incidentalomas (AIs), are observed in computed tomography (CT) at a rate of approximately 3%–8% (1). In recent years, the detection rate of AIs has continued to increase annually with the wide application of CT and magnetic resonance (MR) (2, 3). Most AIs are nonfunctioning adenomas and do not require treatment, and a small proportion is functional adenomas, metastases, and so on, often requiring early clinical intervention. In unenhanced CT, adenomas are often divided into lipid-rich adenoma (≤10 Hounsfield unit, Hu) and lipid-poor adenoma (>10 Hu) at a threshold of 10 Hu (4, 5), the former being easier to differentiate typically due to its presentation (6). However, about 30% of lipid-poor adenomas in adrenal adenomas (7) are difficult to diagnose and often require additional examinations, such as adrenal washout CT, chemical shift MRI, and so on (8, 9). These adenomas have certain disadvantages in that they cannot completely distinguish lipid-poor adenomas, and sometimes the cost of diagnosis and treatment goes waste due to improper response and the risk of additional radiation exposure (10, 11). How to make full use of the information on unenhanced CT, accurately diagnose lipid-poor adenoma, and avoid overdiagnosis and treatment are currently a research hotspot.

Radiomics (12) refers to the extraction of high-dimensional data from medical imaging and has been applied in oncology to improve diagnosis, prediction, and clinical decision support to provide precise medicine. In recent years, some scholars applied radiomics in the diagnosis of adrenal diseases. For example, Moawad et al. (13) used the single-slice method for ROI segmentation task, and the area under the curve (AUC) value of their radiomics model was 0.85, while Zhang et al. (14) used the full-volume method, and its AUC value was 0.91. Although initial progress has been made in the identification of adrenal disease *via* radiomics, whether different ROIs can affect the diagnostic performance of the model still needs further investigation. This study used four different segmentation methods for ROI segmentation to examine the effect of different segmentation methods on radiomics features and model prediction ability.

## 2 Materials and methods

### 2.1 Study participants

The study was conducted according to the Declaration of Helsinki guidelines and was approved by the Ethics Committee of our institution.

The retrospective analysis of clinical imaging data of patients was conducted at institution A (The Affiliated Hangzhou First People’s Hospital, Zhejiang University School of Medicine) and institution B (The Quzhou Hospital of Wenzhou Medical University) from June 2012 to June 2022. The following inclusion criteria were considered: all patients with adrenal lesions underwent unenhanced CT of the abdomen or chest, and the extent of scanning needed to include complete adrenal lesions.

The following exclusion criteria were adopted (1): mean attenuation value of unenhanced CT <10 Hu (2); maximum tumor diameter <1 cm (3); solid component in tumor <50% (4); CT slice thickness >5 mm; and (5) CT poor image quality, *in vitro* foreign body, or motion artifact (6). CT showed that the lesion had been treated with radiotherapy or chemotherapy before the examination (7). When patients had multiple lesions, only the lesion with the largest diameter was analyzed to reduce any clustering effect.

### 2.2 Reference standard

All adrenal lesions ultimately included were pathologically or clinically confirmed (15), and the criterion for clinical confirmation of lipid-poor adenomas was lesion size stability (transverse diameter growth rate <10%) at an imaging follow-up of at least 12 months. The criteria for clinical confirmation of adrenal metastases included new or increased size (30% increase in maximum diameter) of new or existing adrenal lesions within 6 months of imaging follow-up or diagnosis of metastases using positron emission tomography (PET)–CT when the patient had a history of extra-adrenal malignancy. The pheochromocytoma, cortical adenocarcinoma, and schwannoma required pathological confirmation. Solid tumor components were defined as having a CT attenuation value difference greater than 10 Hu before and after enhancement.

**Figure 1:**
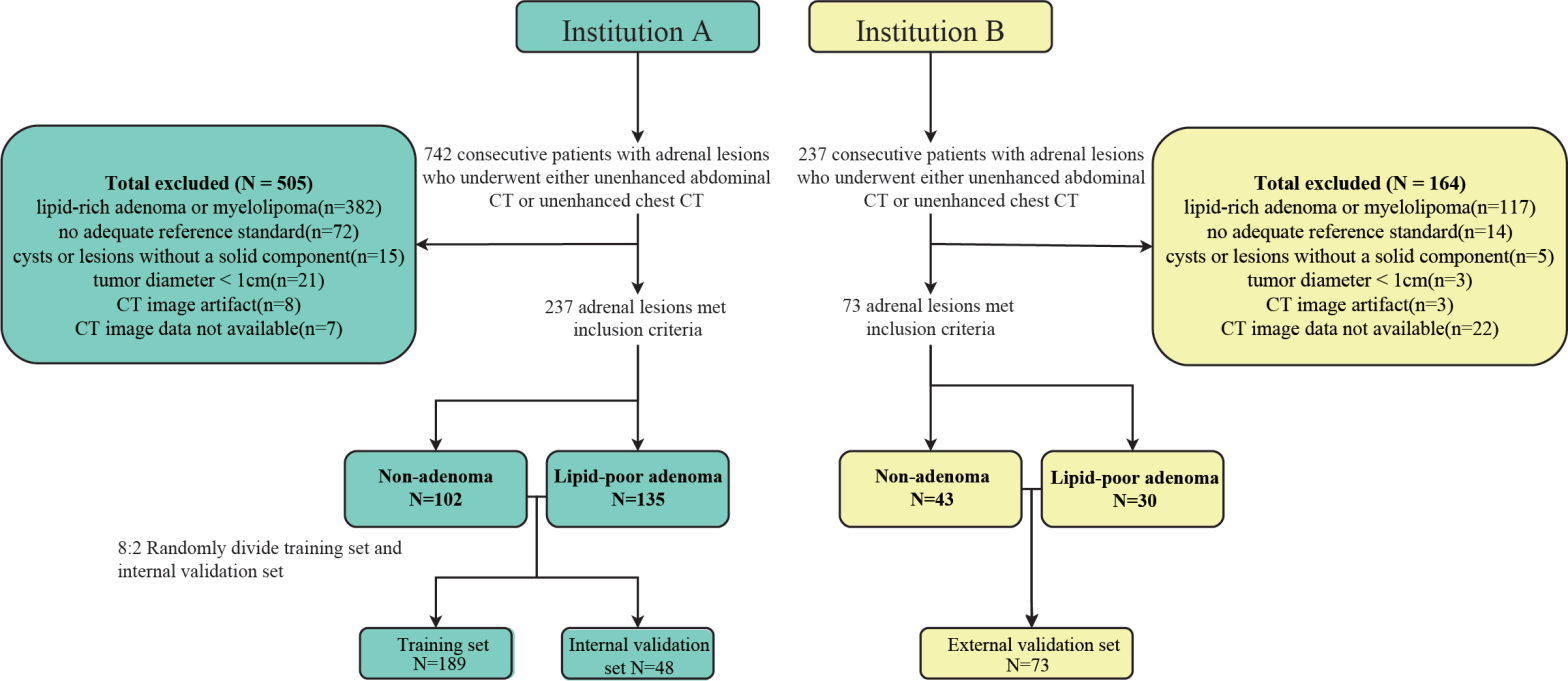
Flowchart of sample inclusion and exclusion in this study.

This study reviewed and analyzed the imaging and clinical data of 979 patients with adrenal lesions at institution A and institution B. A total of 669 patients were excluded, and the imaging data of only 310 patients were included in this study. Out of 310 patients, only 165 patients with lipid-poor adenomas were finally included in this study, as 145 patients were found non-adenomatous. A total of 69 patients with malignant tumor metastasis, 43 with pheochromocytoma, 12 with lymphoma, 11 with ganglioneuroma, 5 with adrenocortical adenocarcinoma, 4 with schwannoma, and 1 with ectopic adrenal accessory spleen were found. In institution A, all 135 patients with lipid-poor adenomas were pathologically confirmed, 64 of 102 patients with non-adenomas were pathologically confirmed, and 38 patients were clinically confirmed. A total of 30 lipid-poor adenomas and 43 non-adenomas were pathologically confirmed in institution B (**Figure 1**).

### 2.3 Image acquisition and conventional imaging analysis

All CT images at institution A were obtained using a Light-speed 16-slice spiral CT system, Revolution 64-slice spiral CT system (General Electric Medical Systems, WI, USA) and uCT 710 62-slice spiral CT system (United Imaging, Shanghai, China). The imaging parameters for unenhanced CT were as follows: 120~140 kV, automated tube current modulation. reconstructed slice thickness was 3.75 mm and 5.00 mm. All CT images at institution B were obtained using an Optima 16-slice spiral CT system (General Electric Medical Systems, WI, USA) and uCT 510 32-slice spiral CT communication system (United Imaging, Shanghai, China), and all patients underwent unenhanced CT using the following imaging parameters: reconstructed slice thickness was 3.75 mm and 5.00 mm, 120~140 kV, automated tube current modulation. All CT image data were uploaded to the Picture Archiving and Communication System (PACS; Radinfo Systems, Zhejiang, China).

The conventional image feature analysis was performed by a radiologist A working for 5 years without knowledge of pathological findings, and lesion diameter, mean attenuation value in unenhanced CT, and lesion location were recorded, as detailed in the **Supplementary Material**.

### 2.4 Radiomics analysis

**Figure 2:**
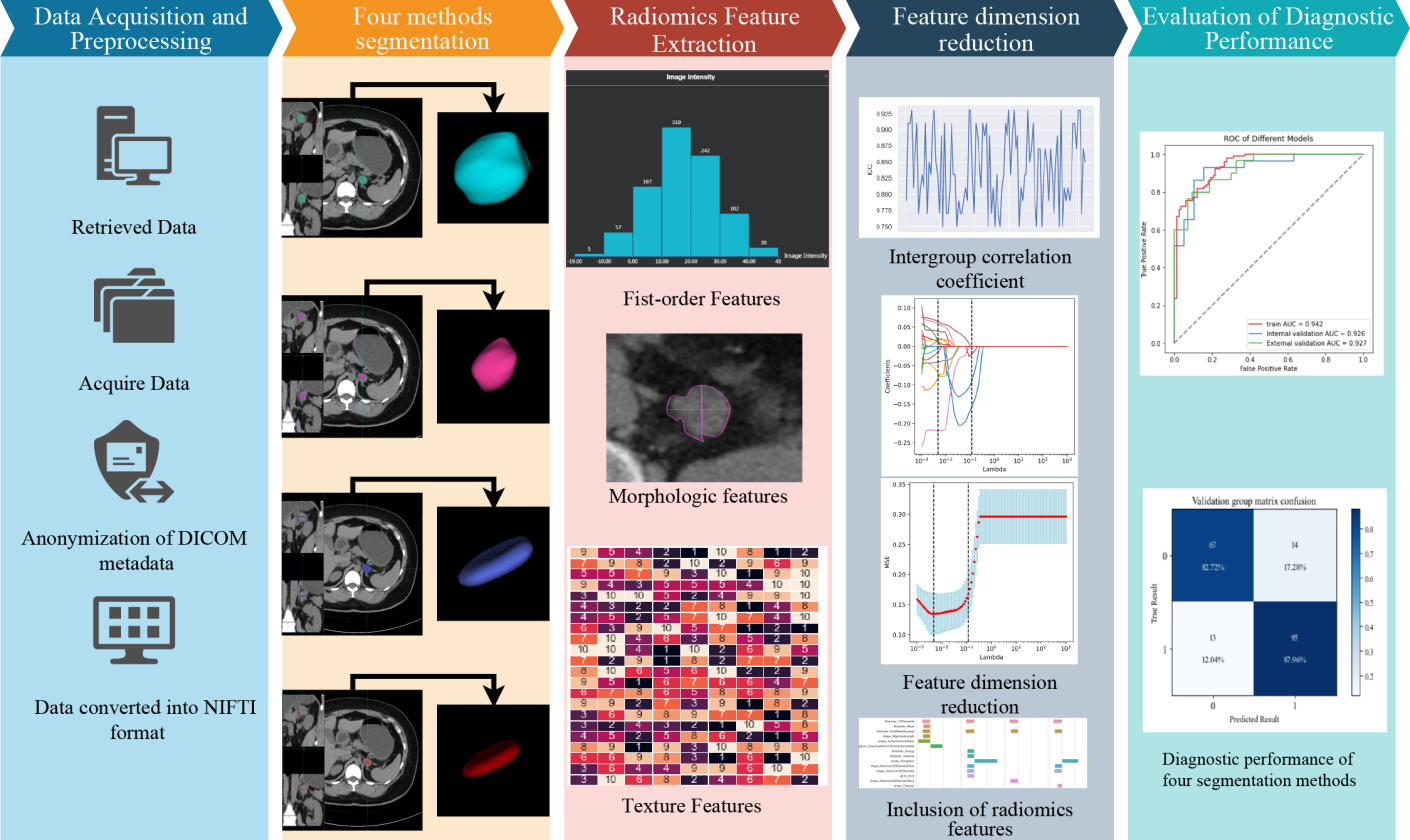
Radiomics analysis flowchart.

The radiomics analysis included data acquisition, desensitization, format conversion, image segmentation, feature extraction, feature selection, and model building. See **Figure 2** for details.

#### 2.4.1 Image segmentation

The ROI segmentation of all images was performed by radiologist A without knowledge of pathological and clinical findings by a semi-automated segmentation method named “Lasso Tool” using uAI Research Portal software (version V1.4; Shanghai United Imaging Company, Shanghai). Semi-automated segmentation methods are detailed in the **Supplementary Material**. The ROI segmentation should avoid obvious cystic degeneration, necrosis, and calcification.

The ROI segmentation is divided into four methods. The first two unmodified segmentation methods are the maximum single-slice segmentation (MAX) and full-volume segmentation (ALL), and the specific operation method of MAX is to select the slice showing the maximum cross-sectional area of the adrenal tumor and segment the ROI along the tumor margin; ALL is the layer-by-layer delineation was performed along the boundary of tumor on CT axial image, and a 3D ROI was generated. In the preliminary experiment of this study, the results indicate that the volume effect diminishes as the ROI gradually shrinks inward. The optimal diagnostic performance of the model is achieved when the ROI is inwardly shrunk by 3mm (Details are in the supplement). The modified single-slice segmentation method (MAX_E): first, the MAX method was used for segmentation, and then the erosion function of the software was used to shrink inward by 3 mm along the *x*- axis and *y*- axis; And the modified full-volume method (ALL_E): segmentation was performed using the ALL method, followed by 3-mm axial shrinkage along *x*-, *y*-, and *z*-axes using the erosion function of the software. The time spent in performing the segmentation task was recorded for the four ROIs.

Four weeks later, a radiologist B with 9-year experience, but no knowledge of the results, randomly selected 50 samples for ROI segmentation again for consistency testing of subsequent radiomics profiles.

#### 2.4.2 Feature extraction and selection

The radiomics features were extracted using uAI Research Portal software. First, the window width window level technique was used to normalize the images, and the “NearestNeighbor” method was used to resample the images, with specific parameters of 3 × 3 × 3 pixel space. In addition, for discrete voxel intensities, bin width was set to 25 HU to reduce image intensity and noise intensity before feature extraction. The extracted radiomics features were mainly divided into three categories: first-order features (FF), morphological features (MF), and texture features (TF). No filter was used for all radiomics features in this study.

The following feature screening steps were considered. First, Stability analysis of radiomics features: The radiomics features with inter-rater correlation coefficient (ICC) <0.75 were excluded. Second, Univariate feature selection: The univariate analysis was performed for the two groups of characteristics to retain the characteristics with *P* < 0.05, and the feature dimension reduction was performed using the recursive feature elimination algorithm to retain the number of features with 30%. Third, Multivariate feature screening: The least absolute shrinkage and selection operator cross-validation for the final feature dimension reduction was performed. The radiomics features were regressed and penalized by tenfold cross-validation and selecting one standard deviation of the minimum lambda.

#### 2.4.3 Construction of radiomics model and evaluation of diagnostic performance

The diagnostic target of this study was lipid-poor adenoma. Logistic regression was used to calculate radiomics scores and in model building. The training set and internal validation set were randomly divided in the ratio of 8:2. The model was established in the training set and internal validation set, and the diagnostic performance was preliminarily assessed. The diagnostic performance and generalization ability of the model were evaluated in an external validation set.

### 2.5 Statistical analysis

The statistical analyses were conducted using R statistical software (version 4.0.3) and Python software (version 3.70). Through the normality test, the continuous variables with normal distribution (age, BMI) were expressed as mean and standard deviation. The continuous variables with non-normal distribution (diameter and unenhanced CT attenuation data) were reported as median (interquartile range, IQR). The categorical variables (sex distribution and CT thickness) were presented as number (%). The normally distributed data were analyzed using the Student *t* test, non-normally distributed data were analyzed using Mann–Whitney *U* test, and categorical variables were analyzed using the chi-square test. Multiple group comparisons were performed using Bonferroni correction. ICCs were used to evaluate the stability of radiomics features extracted by different physicians. The receiver operating characteristic (ROC) curves were drawn to evaluate the diagnostic performance of the models constructed using the four segmentation methods. The evaluation indicators included the AUC and its 95% confidence interval, sensitivity, specificity, and accuracy. Although AUC can assess the entire ROC curve, it is not sufficient to include some areas unrelated to clinical application (such as areas with low specificity) (16). To mitigate this deficit, we calculated a pAUC (partial AUC) with a specificity of at least 95% to distinguish adenomas from non-adenomas because correctly diagnosing benign adenomas in non-enhanced CT has more important clinical implications than misdiagnosing non-adenomas (e.g., metastases), and McNemar’s test was used to compare the sensitivity of the four models. Decision curve analysis (DCA) was used to assess the net benefit of patients in each model. *P* value <0.05 indicated a statistically significant difference.

## 3 Results

### 3.1 Inclusion of baseline and conventional imaging characteristics of patients

The detailed baseline characteristics of patients in the training set (*N* = 189), internal validation set (*N* = 48), and external validation set (*N* = 73) are presented in **Table 1**. The baseline data of all included patients at both institutions comprised 103 women and 62 men in the lipid-poor adenoma group, with a mean age of 53.3 ± 12.5 years, the mean BMI was 24.5 ± 3.4, the median CT attenuation value was 20.2 [14.6;29.4] Hu, and median diameter was 24.0 [16.9;30.0] mm. In the non-adenoma group, 58 women and 87 men were included, with a mean age of 56.3 ± 16.5 years, the mean BMI was 23.5 ± 3.3, the median CT attenuation value was 37.4 [33.2;41.3] Hu, and median diameter was 35.0 [26.3;45.0] mm (**Table 1S**).

**Table 1:** Baseline data of patients in training, internal validation, and external validation sets.

Parameter	Training set (*N* = 189)		Internal validation set (*N* = 48)			External validation set (*N* = 73)			Training set vs internal validation set		Training set vs external validation set	Internal validation set vs external validation set
	LA (*N* = 109)	NA (*N* = 80)		LA (*N* = 26)	NA (*N* = 22)		LA (*N* = 30)	NA (*N* = 43)		*P* value		
Sex^#^										>0.05	>0.05	>0.05
Female	67 (61.47%)	32 (40.00%)		17 (65.38%)	10 (45.45%)		19 (63.33%)	16 (37.21%)				
Male	42 (38.53%)	48 (60.00%)		9 (34.62%)	12 (54.55%)		11 (36.67%)	27 (62.79%)				
Age(year)^*^										>0.05	>0.05	>0.05
Mean (std)	48.8 (9.7)	55.8 (11.9)		57.5 (12.2)	57.2 (20.0)		53.6 (13.0)	56.2 (17.7)				
Distribution^#^										>0.05	>0.05	>0.05
Left	58 (53.21%)	37 (46.25%)		12 (46.15%)	9 (40.91%)		13 (43.33%)	25 (58.14%)				
Right	51 (46.79%)	43 (53.75%)		14 (53.85%)	13 (59.09%)		17 (56.67%)	18 (41.86%)				
BMI^*^										>0.05	>0.05	>0.05
Mean (std)	24.5 (3.5)	23.2 (3.6)		24.7 (3.7)	23.6 (3.3)		24.4 (2.8)	23.9 (2.6)				
Diameter (mm) ^&^										>0.05	>0.05	>0.05
Med [IQR]	25.0 [17.0;30.0]	35.0 [23.6;43.2]		22.6 [17.0;30.0]	32.0 [27.0;40.2]		21.1 [16.3;25.8]	37.8 [31.3;54.4]				
Unenhanced CT Attenuation (Hu) ^&^										>0.05	>0.05	>0.05
Med [IQR]	19.6 [14.6;27.8]	38.6 [34.6;42.8]		23.2 [16.4;31.5]	37.4 [33.1;42.4]		20.7 [14.7;27.1]	35.5 [32.1;37.4]				
Thickness (%) ^#^										>0.05	<0.01	<0.01
3.75 mm	58 (53.21%)	38 (47.50%)		12 (46.15%)	12 (54.55%)		0 (0%)	0 (0%)				
5 mm	51 (46.79%)	42 (52.50%)		14 (53.85%)	10 (45.45%)		30 (100.00%)	43 (100.00%)				

^*^, Student t test; ^&^, Mann–Whitney U test; ^#^, chi-square test; Hu, Hounsfield unit; LA, lipid-poor adenoma; NA, non-adenoma; std, standard deviation. BMI, Body Mass Index; Med, Median; IQR, Interquartile Range.

### 3.2 Characteristics of radiomics in four segmentation methods

**Figure 3:**
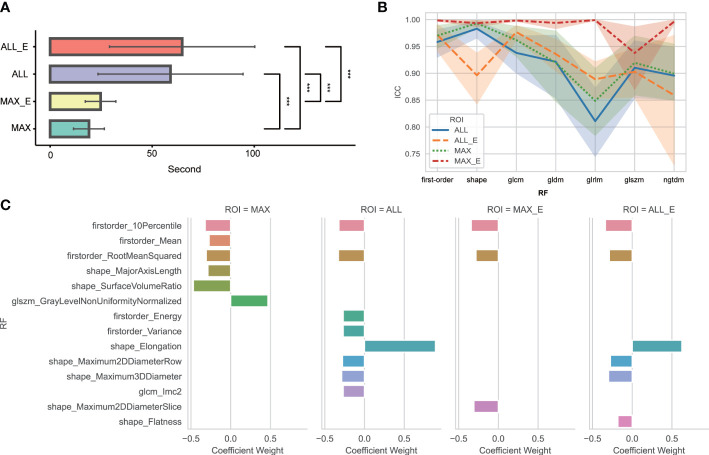
**(A)** Comparative analysis of time consumption between four segmentation methods. ^***^ Indicates *P <*0.01. No statistically significant difference was found between ALL and ALL_E, MAX, and MAX_E. **(B)** ICC variation amplitude for different segmentation methods and radiomics features in each group. glcm is a gray-level co-occurrence matrix; gldm is a gray-level dependence matrix; glrlm is a gray-level run length matrix; glszm is a gray-level size zone matrix; ngtdm is a neighboring gray-tone difference matrix; and RF is radiomics features. **(C)** Radiomics features and weights of the four segmentation methods finally included in the model. RF is radiomics features.

The average time and standard deviation values using MAX, MAX_E, ALL, and ALL_E segmentation methods were 19.0 s ± 7.6, 24.6 s ± 7.6, 58.9 s ± 35.6, and 64.4 s ± 39.6, respectively (**Figure 3A**).

Among 104 unfiltered radiomics features, the FF, MF, and TF features were 18, 14, and 72, respectively. The ICC of MAX, MAX_E, ALL, and ALL_E was 0.934 ± 0.100, 0.987 ± 0.051, 0.919 ± 0.105, and 0.929 ± 0.082, respectively (**Figure 3B**), excluding eight, two, nine, and four radiomics features with ICCs <0.75. After feature dimensionality reduction, the MAX retained six radiomics features, MAX_E retained three radiomics features, ALL retained eight radiomics features, and ALL_E retained six radiomics features (**Figure 3C**).

### 3.3 Comparison of the diagnostic performance of different radiomics models

In all samples, the median and IQR of radiomics scores were calculated for the groups of lipid-poor adenoma and non-adenoma. The median radiomics scores for MAX, MAX_E, ALL, and ALL_E in the lipid-poor adenoma group were 1.5 [0.7; 2.4], 1.8 [0.6; 2.9], 1.9 [0.9; 2.8], and 2.3 [1.0; 3.5], respectively. In the non-adenoma group, the median radiomics scores were 1.2 [0.03; 2.5], 1.7 [0.5; 3.4], 1.7 [0.6; 3.8], and 2.2 [0.7; 4.3]. The pairwise comparisons results showed significant differences between ALL and MAX and between ALL_E and MAX in the lipid-poor adenoma group (*P* = 0.0168 and 0.0002, respectively), and between ALL_E and MAX in the non-adenoma group (*P* < 0.0001). No significant differences were found in the other pairwise comparisons.

**Figure 4:**
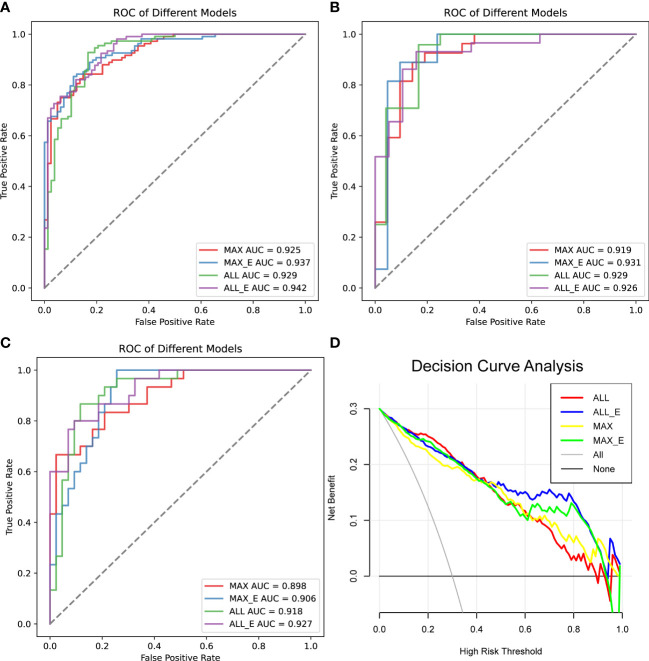
Comparison of the diagnostic performance of the four radiomics models. **(A)** ROC analysis of the training set, **(B)** ROC analysis of the internal test set, and **(C)** ROC analysis of the external validation set. **(D)** Decision curve analysis, the net benefit rate of ALL_E model with High Risk Threshold between 0.5 and 0.8 was the highest. AUC is the area under the curve.

In the training set, the AUCs of MAX, MAX_E, ALL and ALL_E for diagnosing lipid-poor adenoma ranged from 0.925 to 0.942, with sensitivities ranging from 0.889 to 0.914 and specificities ranging from 0.753 to 0.827 (**Figure 4A**, **Table 2S**). The internal validation set showed an AUC range of 0.919 to 0.931, with a sensitivity range of 0.792 to 0.926 and a specificity range of 0.833 to 0.905 (**Figure 4B**, **Table 3S**). The external validation set demonstrated an AUC range of 0.898 to 0.927, with sensitivities ranging from 0.767 to 0.803, and specificities ranging from 0.814 to 0.907 (**Figure 4C**, **Table 2**).

**Table 2:** ROC analysis of four ROI methods for the external validation set.

	AUC	SEN	SPE	ACC
MAX	0.898 [0.829–0.967]	0.767 [0.591–0.882]	0.814 [0.674–0.903]	0.795 [0.688–0.871]
MAX_E	0.906 [0.841–0.972]	0.701 [0.521–0.833]	0.837 [0.700–0.919]	0.781 [0.673–0.860]
ALL	0.918 [0.853–0.983]	0.800 [0.627–0.905]	0.860 [0.727–0.934]	0.836 [0.734–0.903]
ALL_E	0.927 [0.872–0.983]	0.803 [0.627–0.907]	0.907 [0.784–0.963]	0.863 [0.766–0.924]

ACC, Accuracy; AUC, area under curve; SEN, sensitivity; SPE, specificity; 95% confidence interval is within brackets.

In all samples with specificity ≥ 95%, the pAUC values of MAX, MAX_E, ALL, and ALL_E were 0.021, 0.025, 0.018, and 0.028, the sensitivities were 0.545, 0.624, 0.551, and 0.678, and the accuracies were 0.744, 0.770, 0.738, and 0.806, respectively (**Table 3**). There were statistically significant differences between ALL and ALL_E, MAX and ALL_E groups, with *P* values of 0.0243 and 0.0182, respectively. The *P* values for the remaining comparisons are as follows: MAX vs MAX_E (0.1615), MAX vs ALL (0.8330), MAX_E vs ALL (0.1957), and MAX_E vs ALL_E (0.3507). As shown in **Figure 4D**, in DCAs, the net benefit rates of the ALL_E model with High-Risk Threshold between 0.5 and 0.8 were higher than those of the other three models.

**Table 3:** Diagnostic performance of four ROI methods in the differentiation of adenomas from non-adenomas.

	pAUC	SEN	ACC
MAX	0.021 [0.014-0.029]	0.545 [0.418-0.757]	0.744 [0.666-0.847]
MAX_E	0.025 [0.016-0.031]	0.624 [0.515-0.709]	0.770 [0.718-0.821]
ALL	0.018 [0.010-0.028]	0.551 [0.369-0.721]	0.738 [0.641-0.828]
ALL_E	0.028 [0.021-0.035]	0.678 [0.575-0.781]	0.806 [0.751-0.861]

ACC, Accuracy; pAUC, based on partial area under the curve with at least 95% specificity; SEN, sensitivity; SPE, specificity; 95% confidence interval is within brackets.

## 4 Discussion

The main finding of our study was that the ICC of the modified segmentation method was higher than that of the unmodified segmentation method. Furthermore, there was a statistically significant difference in the radiomic score between the model constructed by ALL_E in the improved group and the MAX in the non-improved group. In the external validation set, the diagnostic performance of the radiomics model constructed using ALL was higher than that of MAX, with the AUC of 0.918 and 0.898, respectively, and the diagnostic performance of the modified ALL_E and MAX_E constructed models was better than that of the unmodified ALL and MAX, with the AUC of 0.927 and 0.906, respectively, with ALL_E having the highest AUC on the external validation set. In addition, at least 95% specificity, the number of adenomas diagnosed in the modified MAX_E (103/165 lesions), and ALL_E (112/165 lesions) groups was higher than that in the unmodified MAX (90/165 lesions) and ALL (91/165 lesions) groups, respectively. Compared with the other three methods, ALL_E had the highest number of adenomas diagnosed. Similarly, ALL_E had the highest net clinical benefit rate in DCA. Hence, the radiomics features revealed using ALL_E method could more comprehensively reflect the heterogeneity between adenoma and non-adenoma, and the constructed radiomics model had stronger robustness.

Although radiomics has great potential in the noninvasive and quantitative analyses of lesion image characteristics, it still faces the challenge of clinical translation (17) due to the high magnitude of radiomics feature variation; the segmentation of ROIs is a key step to solve this problem (18). The authors found *via* rigorous literature search significant differences in the selection of ROIs in radiomics studies of adrenal lesions. ROI segmentation methods were mainly divided into single-slice methods and full-volume methods, with the former constructing a diagnostic model with a median AUC of 0.85 (range 0.73–0.97) (13, 19–21) and the latter constructing a model with a median AUC of 0.90 (range 0.80–0.93) (14, 22–25). Numerically, the diagnostic performance of the full-volume method was better than that of the single-slice method, but the patients included in the aforementioned studies came from different medical institutions and the sample baselines were not comparable. Moreover, the sample size included in each study was significantly different, at least 19 cases (22) and at most 292 cases (14). Also, the selection bias and uneven sample size impacted the results so that different ROI segmentation methods used could not quantitatively assess the predictive ability of the model. At present, radiomics research mainly focuses on predicting adrenal diseases (26), and no study has compared and analyzed the effects of different ROIs on radiomics models. In this study, the single-slice and full-volume methods were modified, and the diagnostic performance of the four methods on the external validation set was compared and analyzed. The results showed that the size and location of the ROI were important factors affecting the predictive ability of the model. Theoretically, the full-volume segmentation could cover the tumor more comprehensively (27), and the extracted radiomics features also reflected the FF and MF of the tumor more comprehensively and accurately. The AUC of full-volume method was proved to be slightly higher than that of the single-slice method.

In CT images, the differences in density between adrenal tumors and surrounding organs caused volume confounding effects and reduced the reproducibility of radiomics features and accuracy of the model. We found that there were 13 and 21 more adenomas detected in the modified MAX_E and ALL_E groups, respectively, when specificity was set to at least 95%, compared with the unmodified MAX and ALL groups, and there was a significant statistical difference in sensitivity between ALL_E and ALL, which verified to some extent that volume confounding effects would affect the performance of the model in distinguishing adenomas. Most of the studies conducted so far circumvented the impact of volume effects. Shi et al. (20) and Zhang et al. (14) performed the manual circumvention of lesion margins or removal of the first and last image data of the lesion at ROI segmentation. However, the specific boundary of the volume effect could not be distinguished by the naked eye and could not be completely circumvented by manual segmentation, while removing the image led to the loss of morphological information, resulting in a reduced generalization ability of the model (18). In this study, the conventional method was modified to use a semi-automatic segmentation method and ROI inward contraction of 3 mm to reduce the effect of volume effect and improve the stability of radiomics characteristics, and further increase the sensitivity of the model at high specificity. Therefore, modified ALL_E is more suitable as an ROI segmentation method for constructing radiomics models than unmodified ALL.

However, this study had some limitations. First, in this paper, only the best results of ROI inward contraction of 3 mm are shown, and there is no detailed comparative analysis in the article on the diagnostic performance of ROI inward contraction of 1 mm and 2 mm (see the **Supplementary Material** for the detailed test results). In addition, we did not use ROI inward contraction of 4 mm for segmentation task because large contraction ROIs will lose a large number of valuable texture features, especially in lesions with smaller diameters. Second, the semi-automated segmentation method used in this study was not compared with manual segmentation. Third, the impact of different ROIs on the radiomics model was investigated only *via* unenhanced CT and not *via* enhanced CT. Fourth, the retrospective studies might have some selection bias, besides the small sample size of the external validation set. Hence, further studies should be conducted using a larger sample size.

In conclusion, the selection of ROI is an important factor for reproducing radiomics features and the diagnostic performance of the model in adrenal lipid-poor adenomas. The AUC value of the model constructed by the ALL_E method on the external validation set as well as the pAUC value in the whole sample were the highest. Hence, the radiomics model established by the modified full-volume segmentation method can increase the diagnostic performance and generalization ability of the study results.

## Data availability statement

The raw data supporting the conclusions of this article will be made available by the authors, without undue reservation.

## Ethics statement

The studies involving human participants were reviewed and approved by Affiliated Hangzhou First People’s Hospital, Zhejiang University School of Medicine, and the Quzhou Affiliated Hospital of Wenzhou Medical University. The ethics committee waived the requirement of written informed consent for participation.

## Author contributions

Conceptualization, ZH, HZ, and MW; methodology, HZ, ZH, MW, and MT; software usage and segmentation of ROI, PW and CH; data curation, MT, CH, and TZ; writing original draft preparation, ZH and MW; writing review and editing, ZH and HZ. All authors contributed to the article and approved the submitted version.
