# Supplementary material for: A modified method for CT radiomics region-of-interest segmentation in adrenal lipid-poor adenomas: a two-institution comparative study
Source: Front Oncol. 2023 Apr 19;13:1086039. doi: 10.3389/fonc.2023.1086039 (PMC10154461; doi:10.3389/fonc.2023.1086039)
Supplement: Supplementary file 1 [file DataSheet_1.docx]

Supplementary Material

A modified region-of-interest segmentation method for CT radiomics of adrenal lipid-poor adenoma: a two-institution controlled study

**Hanlin Zhu,^1†^ Mengwei Wu,^2†^ Peiying WEI,^3^ Min Tian,^3^ Tong Zhang,^3^ Chunfeng Hu,^3^ Zhijiang Han^3*^**

Contents

[SUPPLEMENTARY METHODS 3](#_Toc121297234)

[Conventional Imaging Analysis 3](#_Toc121297235)

[Semi-automatic Segmentation Method for Lasso Tool 3](#_Toc121297236)

[SUPPLEMENTARY FIGURE 3](#_Toc121297237)

[Figure 1S 3](#_Toc121297238)

[References 4](#_Toc121297239)

[SUPPLEMENTARY TABLES 5](#_Toc121297240)

[Table 1S 5](#_Toc121297241)

[Table 2S. 6](#_Toc121297242)

[Table 3S. 7](#_Toc121297243)

[RESULTS OF PILOT STUDY 8](#_Toc121297244)

[Table 4S 8](#_Toc121297245)

[Table 5S 8](#_Toc121297246)

[Figure 2S. 9](#_Toc121297247)

.

# SUPPLEMENTARY METHODS

## Conventional Imaging Analysis

The conventional image feature analysis was performed by radiologist A who had 5 years of experience in assessing the image characteristics of the tumor, including tumor diameter, mean CT attenuation value in unenhanced CT, and location of the disease, via the PACS working terminal, without knowledge of the pathological findings. The mean CT attenuation value was measured using the maximum area method: first the largest level of the tumor was selected and then the region of interest (ROI) containing at least two-thirds of the tumor area was manually delineated, avoiding necrotic cystic areas, calcifications, and tumor margin areas during measurement.

## Semi-automatic Segmentation Method for Lasso Tool

The Lasso Tool semi-automatic segmentation method is based on an “intelligent scissor algorithm,” which is an interactive algorithm for image segmentation proposed by Eric N. Mortensen and William A. Barrett in 1995; it can be used for 2D image segmentation(1-2). This algorithm can be used to assist the users to precisely outline the ROI. It can quickly locate to the edge of this image region at runtime, and more accurately complete the delineation and segmentation of the entire ROI through interaction with the user (Figure S1).

# SUPPLEMENTARY FIGURE

| Figure 1S Semi-Automatic Segmentation Example |
| --- |
| 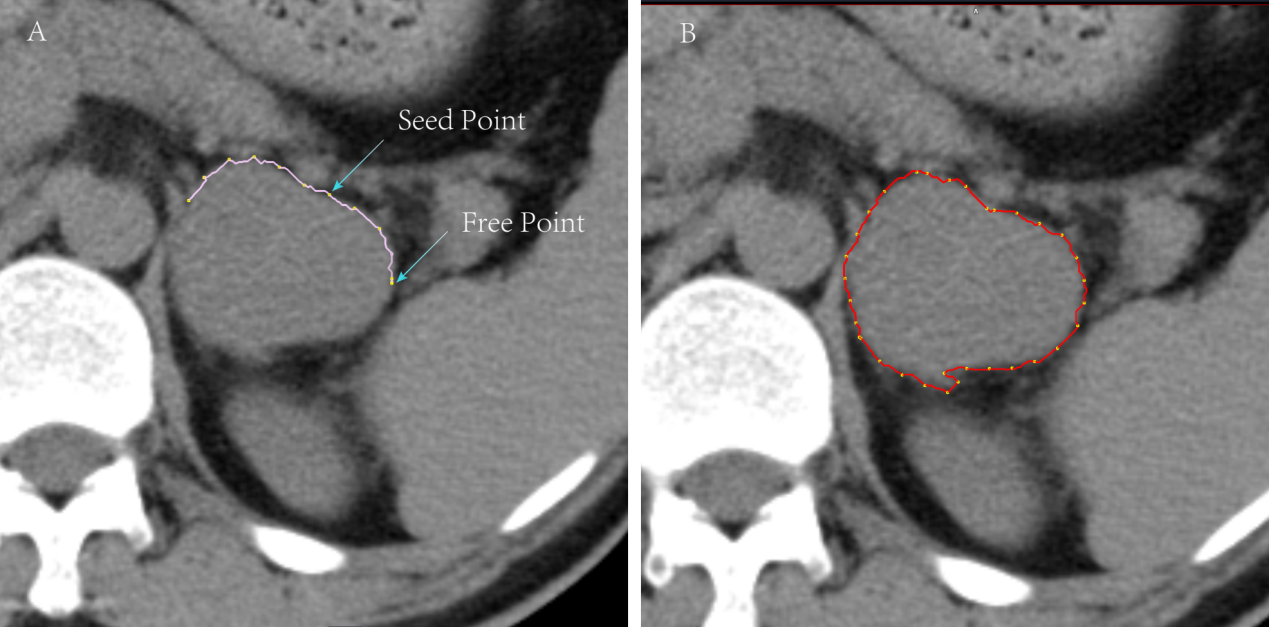 |
| Radiologist A (Fig. S1A) moved the cursor along the edge of the lesion without clicking the mouse. The position of the free point moved as the mouse position changed, automatically generating seed points according to the density difference around the lesion. When delineation was completed, the seed points were automatically merged into the complete ROI (Fig. S1B). |

## References

1. Mortensen E N, Barrett W A. Intelligent scissors for image composition [C]. Proceedings of the 22nd annual conference on Computer graphics and interactive techniques. ACM, 1995:191-198.
2. Mortensen E N, Barrett W A. Interactive segmentation with Intelligent scissors [J]. Graphical models and image processing, 1998, 60 (5): 349-384.

# SUPPLEMENTARY TABLES

| Table 1S**.** Baseline data of patients with lipid-poor adenoma and non-adenoma | | | |
| --- | --- | --- | --- |
| Parameter | **LA**  **(*N* = 165)** | **NA**  **(*N* = 145)** | ***P* value** |
| **Sex^#^** |  |  | **<0.05** |
| **Female** | 103 (62.42%) | 58 (40.00%) |  |
| **Male** | 62 (37.58%) | 87 (60.00%) |  |
| **Age(year) ^*^** |  |  | **<0.05** |
| **Mean (std)** | 53.3 (12.5) | 56.3 (16.5) |  |
| **Distribution (%) ^#^** |  |  | >0.05 |
| **Left** | 83 (50.30%) | 71 (48.97%) |  |
| **Right** | 82 (49.70%) | 74 (51.03%) |  |
| **BMI**^*^ |  |  | **<0.05** |
| **Mean (std)** | 24.5 (3.4) | 23.5 (3.3) |  |
| **Diameter (mm)** ^&^ |  |  | **<0.05** |
| **Med [IQR]** | 24.0 [16.9;30.0] | 35.0 [26.3;45.0] |  |
| **UCT Attenuation (Hu)** ^&^ |  |  | **<0.05** |
| **Med [IQR]** | 20.2 [14.6;29.4] | 37.4 [33.2;41.3] |  |
| **Thickness (%) ^#^** |  |  | >0.05 |
| **3.75 mm** | 71 (43.03%) | 49 (33.79%) |  |
| **5 mm** | 94 (56.97%) | 96 (66.21%) |  |
| ^*^, Student *t* test; ^&^, Mann–Whitney *U* test; ^#^, chi-square test; Hu, Hounsfield unit; LA, lipid-poor adenoma; NA, non-adenoma; std, standard deviation. | | | |

| Table 2S. ROC analysis of four ROI methods for the training set | | | | |
| --- | --- | --- | --- | --- |
|  | AUC | SEN | SPE | ACC |
| MAX | 0.925 [0.89–0.961] | 0.889 [0.816–0.935] | 0.753 [0.649–0.834] | 0.831 [0.771–0.877] |
| MAX_E | 0.937 [0.906–0.968] | 0.889 [0.816–0.935] | 0.827 [0.731–0.894] | 0.862 [0.806–0.904] |
| ALL | 0.929 [0.890–0.967] | 0.914 [0.899–0.981] | 0.795 [0.692–0.870] | 0.881 [0.836–0.926] |
| ALL_E | 0.942 [0.912–0.973] | 0.889 [0.801–0.927] | 0.814 [0.710–0.878] | 0.854 [0.788–0.891] |
| ACC, Accuracy; AUC, area under the curve; SEN, sensitivity; SPE, specificity; 95% confidence interval is within the brackets. | | | | |

| Table 3S. ROC analysis of four ROI methods for the internal validation set | | | | |
| --- | --- | --- | --- | --- |
|  | AUC | SEN | SPE | ACC |
| MAX | 0.919 [0.834–1.000] | 0.926 [0.766–0.979] | 0.810 [0.600–0.923] | 0.875 [0.753–0.941] |
| MAX_E | 0.931 [0.841–1.000] | 0.889 [0.719–0.961] | 0.905 [0.711–0.973] | 0.896 [0.778–0.955] |
| ALL | 0.929 [0.853–1.000] | 0.792 [0.595–0.908] | 0.833 [0.641–0.933] | 0.812 [0.681–0.898] |
| ALL_E | 0.926 [0.851–1.000] | 0.862 [0.694–0.945] | 0.895 [0.686–0.971] | 0.875 [0.753–0.941] |
| ACC, Accuracy; AUC, area under the curve; SEN, sensitivity; SPE, specificity; 95% confidence interval is within the bracket. | | | | |

# RESULTS OF PILOT STUDY

In the initial exploratory trial, we randomly selected 50 lipid-poor adenomas and non-adenomas for testing. We tested the diagnostic performance of 0mm, 1 mm, 2 mm, and 3 mm inward shrinkage of the ROI, respectively. The final test results showed that the optimal diagnostic performance in MAX and ALL methods was an inward contraction of the ROI of 3 mm (Figure S2), and the AUC and AUC with at least 95% specificity were 0.940 and 0.029 (Table S4), 0.953 and 0.034 (Table S5), respectively.

| Table 4S**.** ROC analysis based on MAX method of inward contraction of ROI by 0~3 mm | | | | | |
| --- | --- | --- | --- | --- | --- |
|  | AUC | SEN | SPE | ACC | pAUC |
| 0mm | 0.900 [0.839-0.960] | 0.900 | 0.760 | 0.830 | 0.017 |
| 1mm | 0.917 [0.862-0.973] | 0.920 | 0.820 | 0.870 | 0.021 |
| 2mm | 0.930 [0.878-0.981] | 0.920 | 0.840 | 0.880 | 0.026 |
| 3mm | 0.940 [0.896-0.984] | 0.880 | 0.880 | 0.880 | 0.029 |
| ACC, Accuracy; AUC, area under the curve; SEN, sensitivity; SPE, specificity; 95% confidence interval is within the bracket; pAUC, based on partial area under the curve with at least 95% specificity. | | | | | |

| Table 5S. ROC analysis based on ALL method of inward contraction of ROI by 0~3 mm | | | | | |
| --- | --- | --- | --- | --- | --- |
|  | AUC | SEN | SPE | ACC | pAUC |
| 0mm | 0.942 [0.892-0.992] | 0.920 | 0.880 | 0.900 | 0.015 |
| 1mm | 0.937 [0.893-0.982] | 0.880 | 0.900 | 0.890 | 0.026 |
| 2mm | 0.929 [0.877-0.982] | 0.900 | 0.860 | 0.880 | 0.018 |
| 3mm | 0.953 [0.918-0.988] | 0.880 | 0.900 | 0.890 | 0.034 |
| ACC, Accuracy; AUC, area under the curve; SEN, sensitivity; SPE, specificity; 95% confidence interval is within the bracket; pAUC, based on partial area under the curve with at least 95% specificity. | | | | | |

| Figure 2S. AUC distribution of two segmentation methods |
| --- |
| 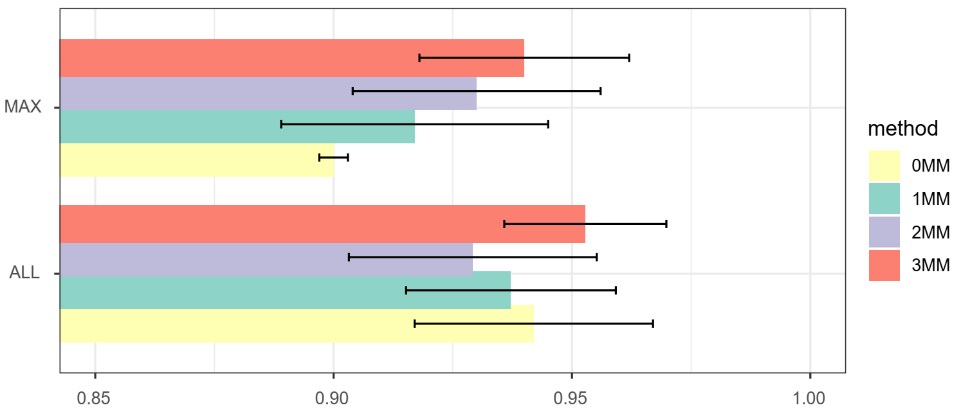 |
| MAX, the maximum area method; ALL, Full Volume Segmentation Method; X-axis, AUC value; Method, Different colors represent 0 mm, 1 mm, 2 mm, and 3 mm inward shrinkage of the ROI. |
